# Acute Human Immunodeficiency Virus Infection Concurrent With Mpox Presenting With Proctitis and Disseminated Skin Lesions: A Case Report

**DOI:** 10.1155/crdi/6286604

**Published:** 2026-08-24

**Authors:** Kosuke Sato, Yukihiro Yoshimura, Yuna So, Nobuyuki Miyata, Yuichiro Hirata, Tadaki Suzuki, Harutaka Katano, Naomi Matsumura

**Affiliations:** ^1^ Department of Infectious Diseases, Yokohama Municipal Citizen’s Hospital, Yokohama, Kanagawa, Japan, yokohama-shiminhosp.jp; ^2^ Department of Infectious Disease Pathology, National Institute of Infectious Diseases, Japan Institute for Health Security, Tokyo, Japan, nih.go.jp; ^3^ Department of Infectious Disease Pathobiology, Graduate School of Medicine, Chiba University, Chiba, Japan, chiba-u.ac.jp; ^4^ Department of Proctology, Coloproctology Center, Matsushima Hospital, Yokohama, Kanagawa, Japan

**Keywords:** acute HIV infection, anorectal involvement, case report, men who have sex with men, mpox, proctitis

## Abstract

**Background:**

The concurrent presentation of mpox and acute human immunodeficiency virus (HIV) infection is uncommon and may be difficult to recognize because the two conditions share nonspecific early manifestations, including fever, rash, lymphadenopathy, and pharyngeal symptoms. Mpox‐associated anorectal disease may further complicate diagnosis when patients initially present with severe anal pain or proctitis‐like findings.

**Case Presentation:**

A previously healthy 29‐year‐old Japanese man presented with fever, malaise, severe anal pain, and disseminated pustular skin lesions. He initially visited a specialized proctology hospital, where lower endoscopic examination demonstrated circumferential ulceration extending from the dentate line into the anal canal, erythematous and edematous rectal mucosa, and firm circumferential submucosal induration. The anorectal lesion was initially considered to represent a hemorrhoidal fistula. He subsequently visited the emergency department and received valacyclovir for suspected herpes simplex virus infection before referral to the infectious diseases department. Mpox was confirmed by polymerase chain reaction testing of skin lesions. In addition, monkeypox virus DNA was detected in the anorectal biopsy specimen, and immunohistochemical staining demonstrated viral antigen in the tissue. HIV testing showed a reactive fourth‐generation antigen/antibody screening assay, a negative HIV‐1/2 antibody differentiation assay, and a plasma HIV RNA level of 7.9 × 10^6^ copies/mL. Follow‐up testing demonstrated HIV seroconversion, confirming acute HIV infection. He was managed as an outpatient, and his mpox symptoms resolved with supportive care. Antiretroviral therapy was subsequently initiated, with no worsening of mpox or evidence of immune reconstitution inflammatory syndrome.

**Conclusion:**

Concurrent acute HIV infection and mpox can present with overlapping systemic, cutaneous, and anorectal manifestations. This case highlights the importance of integrating lesion morphology, anorectal findings, epidemiological history, and virological testing when evaluating patients with suspected sexually transmitted viral infections.

## 1. Introduction

Mpox is a zoonotic disease caused by the monkeypox virus (MPXV) that has re‐emerged as a global public health concern since the multinational outbreak in 2022 [1]. Although the number of reported cases has declined from the initial epidemic, ongoing transmission continues, particularly among men who have sex with men (MSM) [1, 2]. Because transmission frequently occurs through close physical and sexual contact, coinfection with sexually transmitted infections, including human immunodeficiency virus (HIV), is commonly encountered [1, 2]. In Japan, the first case of mpox was reported on July 25, 2022, and sporadic domestic cases have continued to be reported since then [3].

Most reported cases of HIV‐associated mpox have occurred in people living with established HIV infection receiving routine HIV care [2, 4]. In contrast, concurrent presentation of mpox and acute HIV infection has rarely been described [5, 6]. Acute HIV infection often presents with nonspecific symptoms, such as fever, rash, lymphadenopathy, and pharyngitis, which substantially overlap with those of mpox [1, 7]. During acute HIV infection, plasma HIV RNA becomes detectable before HIV‐specific antibodies develop [7]. Therefore, virological testing plays a key role in establishing the diagnosis during the early phase of infection. These overlapping clinical and laboratory features may delay recognition of either disease, particularly in settings where clinicians have limited experience with mpox.

We report a case of concurrent acute HIV infection and mpox in a previously healthy Japanese MSM, in whom diagnosis was delayed because of overlapping clinical manifestations and initial assessment in nonspecialist settings.

## 2. Case Presentation

Ethical approval was not required for this case report in accordance with the policy of our institution. Written informed consent was obtained from the patient for publication of this report and any accompanying images.

### 2.1. History of Present Illness

**FIGURE 1:**
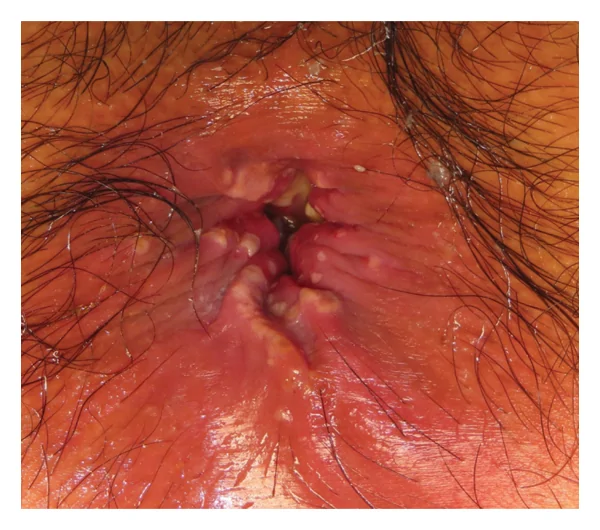
Anal lesion showing an ulcerative lesion with erythematous base and surrounding edema. The lesion appeared painful and indurated, with superficial erosion and exudate at the margin.

The patient was a previously healthy 29‐year‐old Japanese man with no significant medical history and no regular medications. He smoked 20 cigarettes per day and consumed approximately 1000 mL of beer daily. He had no history of smallpox vaccination. Ten days before presentation to our department, he developed a fever and malaise. Three days later, he noticed papular skin lesions on his extremities, accompanied by worsening anal pain. He first sought medical attention at a specialized proctology hospital because of severe anal pain. An anorectal examination was subsequently performed under spinal anesthesia. The patient had a circumferential ulcer extending from the dentate line into the anal canal, with partial extension beyond the anal canal. The rectal mucosa was erythematous and edematous. Circumferential submucosal induration was palpable. A biopsy specimen was obtained from the inflammatory lesion at the anorectal mucocutaneous junction. Routine pathological examination of the biopsy specimen initially suggested a hemorrhoidal fistula. The anorectal lesion at the initial presentation to the proctology hospital is shown in Figure 1. A preprocedural HIV screening test was reactive, and he was referred to the department of infectious diseases. However, his fever persisted, and the skin lesions continued to worsen. Eight days after his initial visit to the proctology hospital, he presented to the emergency department. Valacyclovir was prescribed because herpes simplex virus infection was suspected. Two days later, he visited the department of infectious diseases for further evaluation of the skin lesions and HIV screening results.

During the infectious diseases consultation, additional history revealed that the patient was a MSM. He had a regular male sexual partner for several months and also reported sexual contact with other partners whom he met through a social networking application approximately once every 2 months. All reported sexual partners were Japanese. The last sexual contact had occurred approximately 1‐2 weeks before symptom onset. The patient had no history of overseas travel. Based on the clinical findings and sexual history, mpox was suspected.

### 2.2. Physical Examination

**FIGURE 2:**
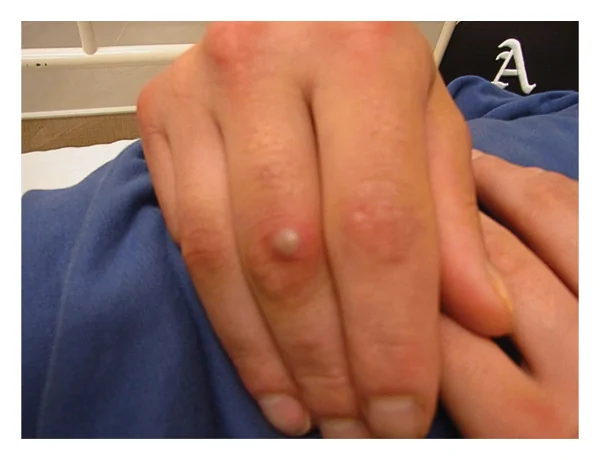
Cutaneous lesions on the patient’s hands: multiple papules with central pustules are visible on both the palmar and dorsal aspects of the hands.

Vital signs were as follows: The patient was alert. Respiratory rate was 16 breaths/min, body temperature was 38.4°C, pulse rate was 114 beats/min, blood pressure was 116/66 mmHg, and SpO_2_ was 100% on room air. His face was flushed, and mild pharyngeal erythema was present. Cervical lymphadenopathy was present without tenderness. There were no abnormal chest or abdominal findings. The patient presented with firm papules accompanied by central pustules on the anterior neck and thighs. Similar pustular papules were also observed on both hands (Figure 2).

### 2.3. Laboratory Findings

Selected laboratory findings at initial presentation are shown in Table 1. The fourth‐generation HIV antigen/antibody screening assay was reactive, whereas the HIV‐1/2 antibody differentiation assay was negative. Plasma HIV RNA was 7.9 × 10^6^ copies/mL, and the CD4‐positive lymphocyte count was 307 cells/mm^3^. Tests for β‐D‐glucan, rapid plasma reagin, and *Treponema pallidum* antibodies were negative.

**TABLE 1:** Laboratory findings at initial presentation.

Laboratory parameter	Result	Reference range
White blood cell count (/μL)	5590	3300–8600
Neutrophil percentage (%)	61.5	40–70
Lymphocyte percentage (%)	28.8	20–45
C‐Reactive protein (mg/dL)	19.2	< 0.30
Fibrinogen (mg/dL)	503	200–400
D‐dimer (μg/mL)	1.99	< 1.0
Aspartate aminotransferase (U/L)	40	13–30
Alanine aminotransferase (U/L)	40	10–42
CD4‐positive lymphocyte count (cells/mm^3^)	307	500–1500
Fourth‐generation HIV antigen/antibody assay	30.66 (reactive)	Non‐reactive
Plasma HIV RNA (copies/mL)	7.9 × 10^6^	Not detected
HIV‐1/2 antibody differentiation assay	Negative	Negative
Rapid plasma reagin test	0.8	< 1.0
*Treponema pallidum* antibody	0.02	< 10
Herpes simplex virus IgG	4.3	< 0.8
Herpes simplex virus IgM	1.23	< 0.8
Varicella‐zoster virus IgG	39.4	< 0.8
Varicella‐zoster virus IgM	0.31	< 0.8

Abbreviation: HIV, human immunodeficiency virus.

The pustular contents on his hands were submitted to the city health laboratory for real‐time polymerase chain reaction (PCR) testing. The following day, real‐time PCR analysis of the blister fluid confirmed MPXV infection, establishing the diagnosis of mpox. After mpox was diagnosed, the anorectal biopsy specimen underwent additional pathological and virological evaluation.

Nucleic acids were extracted from formalin‐fixed, paraffin‐embedded (FFPE) anal tissue at the National Institute of Infectious Diseases using the Quick DNA/RNA FFPE MiniPrep, and MPXV DNA was quantified by real‐time PCR. Beta‐actin was amplified as an endogenous control. MPXV DNA (812,650 copies/μL) and beta‐actin (106,500 copies/μL) were detected in the anal tissue specimens. Clade typing was also performed, leading to the identification of the virus as Clade II.

**FIGURE 3:**
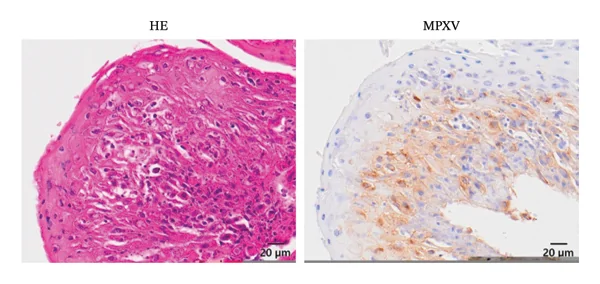
Histopathological and immunohistochemical analysis of the perianal lesion: hematoxylin and eosin (HE) staining was performed, and additional immunostaining using an anti‐vaccinia virus LC16m8 antibody as the primary antibody demonstrated positive monkeypox virus (MPXV) antigen expression in the anal tissue.

Immunohistochemical staining using an anti‐vaccinia virus LC16m8 antibody demonstrated positive staining for MPXV in the anal tissue specimen (Figure 3).

### 2.4. Clinical Course

The patient was managed as an outpatient with close follow‐up and received acetaminophen for symptomatic relief. Tecovirimat was not administered because it was not available for routine clinical use in Japan at the time and because the patient did not have severe or progressive mpox warranting investigational antiviral therapy. The fever resolved approximately 2 weeks after symptom onset, and systemic symptoms, including headache and malaise, subsided. All skin lesions had crusted by approximately 3 weeks after symptom onset.

**FIGURE 4:**
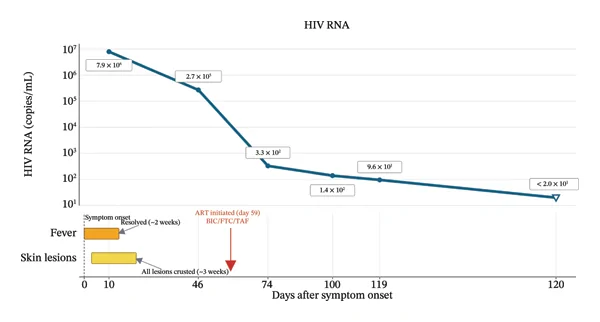
Clinical course of the patient with acute HIV infection and MPXV coinfection: timeline shows the onset of perianal lesion and fever, progression of rash, laboratory findings including HIV RNA viral load, and initiation of antiretroviral therapy (ART).

Follow‐up HIV testing demonstrated seroconversion, confirming the diagnosis of acute HIV infection. Antiretroviral therapy with bictegravir, emtricitabine, and tenofovir alafenamide (BIC/TAF/FTC) was initiated 7 weeks after the patient’s initial visit, following evaluation for concomitant infections and other clinical complications. Thereafter, the plasma HIV RNA level decreased, and the patient continued outpatient follow‐up without worsening of mpox or evidence of immune reconstitution inflammatory syndrome (Figure 4).

## 3. Discussion

This case illustrates the diagnostic difficulty of concurrent acute HIV infection and mpox. The major challenge was that both conditions can present with overlapping early manifestations, including fever, rash, lymphadenopathy, and pharyngeal symptoms, making clinical distinction based on the initial presentation alone difficult. In the present case, the morphology of the skin lesions, anorectal involvement, epidemiological background, and virological test results were all required to establish the diagnoses. This case emphasizes the importance of integrating clinical findings, sexual history, lesion distribution, and virological testing when evaluating patients with suspected sexually transmitted viral infections.

The clinical overlap between acute HIV infection and mpox was central to the diagnostic difficulty in this case. Acute HIV infection may present with nonspecific systemic symptoms, including fever, lymphadenopathy, pharyngitis, rash, myalgia, and arthralgia [7]. Mpox can also present with fever, lymphadenopathy, sore throat, malaise, and rash, and lesions may involve the genital, anal, or perianal regions [1, 2]. In our patient, fever, malaise, mild pharyngeal redness, cervical lymphadenopathy, and disseminated skin lesions could not be attributed confidently to either acute HIV infection or mpox on clinical grounds alone. Therefore, virological testing was essential for distinguishing and confirming both conditions.

The morphology of the skin lesions provided an important diagnostic clue in this patient. Although fever, malaise, lymphadenopathy, and pharyngeal symptoms were nonspecific, the presence of firm papules with central pustules raised clinical suspicion for mpox. This suspicion was further supported by the distribution of the lesions, the presence of anorectal disease, and the epidemiological history obtained during the infectious diseases consultation. However, the clinical appearance alone was not sufficient for diagnosis [8], and other conditions, including herpes simplex virus infection and eosinophilic pustular folliculitis associated with acute HIV infection, were also considered during the clinical course. The diagnosis of mpox was ultimately confirmed by MPXV PCR testing of the skin lesions.

Anorectal involvement was another important feature of this case. During the 2022 mpox outbreak, anorectal pain, proctitis, tenesmus, rectal bleeding, and ulcerative anal or perianal lesions were increasingly recognized as clinical manifestations of sexually acquired mpox [6, 9–11]. In our patient, lower endoscopic examination at the proctology hospital demonstrated circumferential ulceration extending from the dentate line into the anal canal, with partial extension outside the anal canal. The rectal mucosa was erythematous and edematous, and circumferential firm submucosal induration was palpable. A biopsy was obtained from the anorectal mucosal transition zone. Detection of MPXV DNA by PCR and positive immunohistochemical staining for MPXV antigen in the biopsy specimen strongly supported direct MPXV involvement of the anorectal lesion. Therefore, the patient’s severe anal pain and proctitis‐like findings were most consistent with mpox‐associated anorectal disease.

The diagnosis of acute HIV infection was supported by both virological and serological findings. The fourth‐generation HIV antigen/antibody screening assay was reactive, whereas the HIV‐1/2 antibody differentiation assay was negative. In addition, plasma HIV RNA was markedly elevated at 7.9 × 10^6^ copies/mL. This pattern is consistent with acute HIV infection, in which plasma HIV RNA becomes detectable before the development of HIV‐specific antibodies [7]. Follow‐up testing subsequently demonstrated HIV seroconversion, further supporting the diagnosis of acute HIV infection [7].

The timing of ART initiation also requires discussion. Current guidance recommends early initiation of ART in people with newly diagnosed HIV infection, including those with mpox, because the benefits of viral suppression generally outweigh concerns regarding immune reconstitution inflammatory syndrome [7, 12]. In the present case, ART was initiated 7 weeks after the patient’s visit. This delay reflected the clinical context at that time, including limited institutional experience with mpox, careful observation of the natural course of mpox‐associated skin and anorectal lesions, and evaluation for concomitant infections and other complications before treatment. In retrospect, this case supports the importance of early linkage to HIV care and timely ART initiation in patients with concurrent mpox and newly diagnosed acute HIV infection.

## 4. Conclusion

Concurrent acute HIV infection and mpox may present with overlapping systemic, cutaneous, and anorectal manifestations, making early diagnosis challenging. Severe anal pain or proctitis‐like findings accompanied by pustular skin lesions should prompt consideration of mpox, particularly in patients with epidemiological risk factors. This case emphasizes the importance of integrating clinical findings, sexual history, lesion distribution, anorectal evaluation, and virological testing when evaluating patients with suspected sexually transmitted viral infections.

## Funding

This work was supported in part by the Japan Science and Technology Agency (JST), Moonshot R&D (JPMJMS2025), the Japan Agency for Medical Research and Development (AMED) (JP223fa627003, JP26fk0108732), and the Emerging and Re‐emerging Infectious Diseases and Vaccination Policy Promotion Research Project of the Ministry of Health, Labour and Welfare, Japan (26HA2015).

## Disclosure

All authors contributed to the preparation of the final manuscript, approved the final version for publication, and agree to be accountable for all aspects of the work, including the accuracy, integrity, content, and conclusions of the article.

## Conflicts of Interest

The authors declare no conflicts of interest.

## Data Availability

The data supporting the findings of this case report are available from the corresponding author upon reasonable request. To protect patient privacy and confidentiality, individual‐level data are not publicly available.
